# miR-142-3p as a biomarker of blastocyst implantation failure - A pilot study

**DOI:** 10.5935/1518-0557.20160039

**Published:** 2016

**Authors:** Edson Borges Jr., Amanda Souza Setti, Daniela P.A.F. Braga, Murilo V Geraldo, Rita de Cássia S Figueira, Assumpto Iaconelli Jr

**Affiliations:** 1 Fertility Medical Group - São Paulo/SP - Brazil; 2 Instituto Sapientiae - Centro de Estudos e Pesquisa em Reprodução Assistida - São Paulo/SP - Brazil; 3 Disciplina de Urologia, Área de Reprodução Humana, Departamento de Cirurgia, Universidade Federal de São Paulo/SP - Brazil; 4 Departamento de Biologia Estrutural e Funcional, Instituto de Biologia, Universidade Estadual de Campinas UNICAMP/SP - Brazil

**Keywords:** Blastocyst, culture medium, ICSI, implantation, MicroRNA

## Abstract

**Objective:**

This study aims to find whether microRNAs (miRNAs) detected in the culture medium of embryos produced in vitro could be potential biomarkers of embryo implantation.

**Methods:**

Culture media samples from 36 embryos, derived from patients undergoing intracytoplasmic sperm injection (ICSI) in a private university-affiliated IVF center, were collected between January/2015 and November/2015. Samples were collected on day three and embryo transfers were performed on day five and all embryos reached the blastocyst stage. Samples were split into groups according to the embryo implantation result: Positive-Implantation-Group (n=18) or Negative-Implantation-Group (n=18). For the first analysis, samples were pooled in three sets for each group (6-7 spent media per pool). MicroRNAs were extracted from spent media and cDNA was synthesized. C. elegans *miR-39* was used as RNA spike-in to normalize the gene expression analysis. The expression of microRNAs into the spent media from the Positive-Implantation-Group was compared with those from the Negative-Implantation-Group. A set of seven miRNAs (*miR-21, miR-142-3p, miR-19b, miR-92a, miR-20b, miR-125a and miR148a*) selected according with the literature, was tested. To check whether miRNAs could be detected in individual samples of culture media, in a second analysis, ten more samples were tested for *miR-21* and *miR-142-3p*.

**Results:**

From the sevens tested miRNAs, a significant increased expression of miR-142-3p could be noted in the Negative-Implantation-Group (*P*<0.001). For other three miRNAs (*miR-21, miR-19b* and *miR-92a*) a difference in expression was observed, however it did not reach a statistical significance. In addition, when ten non-redundant samples were tested to check if miRNAs could be detected in individual samples of culture media, the highly specific amplification of mature miRNAs, including *miR-142-3p*, could be noted.

**Conclusion:**

Our findings suggest that *miR-142-3p*, previously described as a tumor suppressor and cell cycle inhibitor, may be a potential biomarker of blastocyst implantation failure. The identification of miRNAs on individual culture medium samples offers unique opportunities for non-invasive early diagnosis of blastocyst implantation.

## INTRODUCTION

The use of assisted reproductive technology (ART) has dramatically increased in the past decades. Despite the technical progress achieved in embryo culture and areas such as culture medium and incubators, most transferred embryos fail to implant (de Mouzon *et al*., 2012). Multiple-embryo transfers are commonly performed to compensate for the relatively low efficiency of the procedure. However, this practice often results in multiple pregnancies (Pandian *et al*., 2009; Setti & Bulletti, 2011), an undesired outcome that occurs thirty times more frequently in women undergoing ART than in women with spontaneous pregnancies (ACOG, 2005). Single-embryo transfer (SET) may reduce the rate of multiple pregnancies. The success of SET relies on the optimal selection of a single embryo for transfer, based on morphologic criteria.

Optimal embryo selection for transfer is challenging. The ability of the several scoring systems available today to assess embryo potential seems to have reached a plateau. Thus, it is of interest to discover a biomarker of embryo viability and implantation potential that leads to higher pregnancy rates while reducing the number of multiple pregnancies via SET (Rosenbluth *et al*., 2014). An ideal biomarker should allow non-invasive embryo assessment based on the analysis of the surrounding culture medium. Many potential embryo biomarkers have been recently investigated. Secreted proteins and metabolites were identified in embryo culture medium (Cortezzi *et al*. 2011; Hardarson *et al*. 2012; Vergouw *et al*., 2012; Cortezzi *et al*., 2013); however, this technology has not led to an improved ability to predict embryo implantation potential.

More recently, the role of microRNAs (miRNAs) in embryo development and implantation has been investigated (Suh & Blelloch, 2011). MiRNAs are endogenous, evolutionally conserved, single-strand non-coding RNA molecules of 20-24 nucleotides, that post-transcriptionally regulate gene expression in eukaryotes, including mammalian cells (Asirvatham *et al*., 2009; McCallie *et al*., 2010; Mouillet *et al*. 2015; Thouas *et al*. 2015). They were first described in the nematode Caenorhabditis elegans (Lee *et al*. 1993; Wightman *et al*. 1993) and later found in the genomes of protists, plants, animals, and viruses (Mouillet *et al*., 2015). In humans, miRNAs have been detected in virtually all bodily fluids, including blood, urine, saliva, tears, breast milk, semen, amniotic fluid, cerebrospinal fluid, peritoneal fluid, and pleural fluid as well as in culture medium collected from different cell lines (Wang *et al*., 2010a; Wang *et al*., 2010b; Weber *et al*., 2010).

Currently, more than 2,500 human miRNAs are listed in the biological database miRBase (http://mirbase.org). They are believed to be involved in virtually every biological process, modulating regulatory pathways that control early embryo development (Laurent, 2008), cell growth (Carleton *et al*., 2007), development (Tang *et al*., 2007) and differentiation (Lakshmipathy *et al*., 2007) and organ function in health and disease, including several types of cancers (Barbarotto *et al*., 2008), viral infections (Sullivan & Ganem, 2005), and heart disease (Tatsuguchi *et al*., 2007).

MiRNAs have been shown to play an important role during mouse embryonic development, with an overall surge toward the blastocyst stage (Yang *et al*., 2008). More than 130 miRNAs are expressed in the human blastocyst (McCallie *et al*., 2010; Rosenbluth *et al*., 2013). McCallie *et al.,* (2010) first described that blastocyst derived from infertile patients have atypical miRNA profiles. Later, it was demonstrated that miRNA expression in blastocysts differs between euploid and aneuploid embryos, as well as between genders (Rosenbluth *et al*., 2013). Specific miRNAs are also detectable in spent blastocyst culture medium, with correlations to oocyte insemination method, embryo ploidy, and live birth (Rosenbluth *et al*., 2014). Recently, Capalbo *et al.* (2016) have comprehensively characterized the population of miRNAs secreted from human blastocysts into spent culture medium, and two miRNAs (miR-20a, miR-30c) were positively correlated with blastocyst implantation.

Still, very few studies have investigated the correlation between miRNA expression and embryo implantation potential. The objective of this study was to identify miRNAs secreted by embryos in culture medium that could be potential biomarkers of blastocyst implantation.

## MATERIAL AND METHODS

### Study Design

This pilot study included spent culture medium from 36 embryos, derived from patients undergoing intracytoplasmic sperm injection (ICSI) in a private university-affiliated IVF center, collected between January/2015 and November/2015. The samples were collected on day three and the embryo transfer procedures were performed on day five; all embryos reached the blastocyst stage. The samples were split into groups with positive (n=18) or negative (n=18) implantation outcomes. In the first analysis, the samples were pooled in three sets for each group. The positive and negative implantation groups were compared for microRNA expression in the spent medium. A set of seven miRNAs, selected according to the literature, was tested. Ten additional samples were tested for miRNAs in individual samples of culture medium in a second analysis cycle.

The patients consented in written to having their cycle outcomes analyzed in this study. The local institutional review board approved the study.

### Controlled ovarian stimulation

Controlled ovarian stimulation (COS) was achieved by pituitary blockage using a GnRH antagonist (Cetrotide; Serono, Geneva, Switzerland); and ovarian stimulation was performed using recombinant FSH (Gonal-F; Serono, Geneva, Switzerland).

Follicular growth was monitored using transvaginal ultrasound examination starting on day four of gonadotropin administration. When adequate follicular growth and serum estradiol levels were observed, recombinant hCG (Ovidrel; Serono, Geneva, Switzerland) was administered to trigger final follicular maturation. The oocytes were collected 35 hours after hCG administration through transvaginal ultrasound-guided ovum pickup.

### Oocyte preparation

The retrieved oocytes were maintained in culture medium (Global® for fertilization, LifeGlobal, Connecticut, USA) with 10% protein supplement (LGPS, LifeGlobal) and covered with paraffin oil (Paraffin oil P.G., LifeGlobal) for two to three hours before the removal of cumulus cells. The surrounding cumulus cells were removed after exposure to a HEPES-buffered medium containing hyaluronidase (80 IU/mL, LifeGlobal). The remaining cumulus cells were mechanically removed gently by pipetting with a hand-drawn Pasteur pipette (Humagen Fertility Diagnostics, Charlottesville, USA).

Oocyte morphology was assessed using an inverted Nikon Diaphot microscope (Eclipse TE 300; Nikon^®^, Tokyo, Japan) with a Hoffmann modulation contrast system under 400X magnification just before sperm injection (4 hours after retrieval). The oocytes observed to have released the first polar body were considered mature and were used for ICSI.

### IVF procedures and spent medium collection

Intracytoplasmic sperm injection was performed in a micro-injection dish prepared with 4 µL droplets of buffered medium (Global^®^ w/HEPES, LifeGlobal) and covered with paraffin oil on the heated stage of an inverted microscope at 37.0 ± 0.5°C. Approximately 16 hours after ICSI, fertilization was confirmed by the presence of two pronuclei and the extrusion of the second polar body. The embryos were maintained in a 50 µL drop of culture medium (Global^®^, LifeGlobal) with 10% protein supplement and covered with paraffin oil in a humidified atmosphere under 6% CO2 at 37ºC for three days. The embryos were moved to fresh medium droplets and were cultured until day-5 of development; 20µL of the spent medium were collected and stored at -80º C for miRNA analysis. One or two embryos were transferred on day five.

### miRNA Isolation and Detection

To maximize the total amount of RNA available from each spent medium sample collected, cDNA was synthesized using the Taqman MicroRNA Reverse Transcription Kit (Life Technologies, Carlsbad, CA, USA) according to manufacturer instructions. C. elegans miR-39 was used as an RNA spike-in to normalize gene expression analysis. The detection of miRNAs was performed using Taqman miRNA Assays (Life Technologies). The analysis of the expression obtained in real-time quantitative PCR was performed using the SDS software (Life Technologies).

In a further step, cDNA was individually synthesized for each tested miRNA using the miRNA Reverse Transcription kit (Life Technologies), according to manufacturer instructions. The detection of miRNA expression was performed by quantitative real-time PCR, using the TaqMan^®^ MiRNA Assay system (Life Technologies).

### Statistical analyses

Comparisons between experimental groups were performed using the ΔΔCt method. The statistical significance of fold changes was determined by performing an unpaired, two-tailed Mann-Whitney test of the ΔΔCt values.

## RESULTS

**Figure 1:**
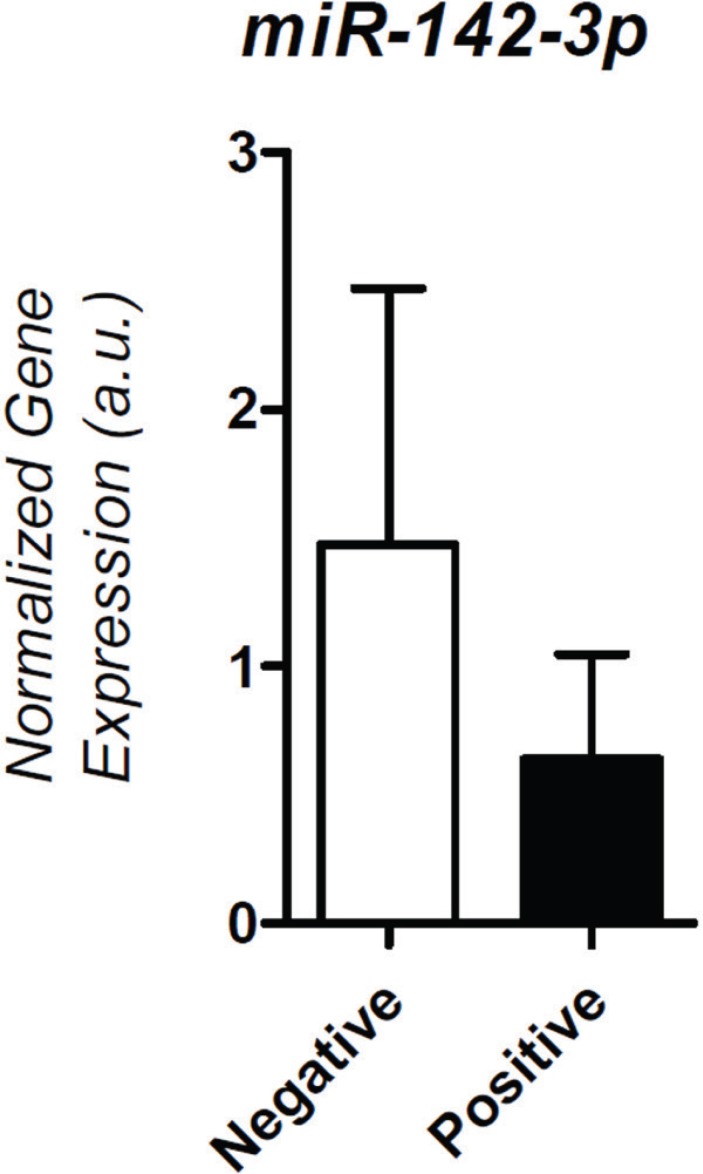
Expression of miR-142-3p in the Positive and Negative Implantation groups.

**Figure 2:**
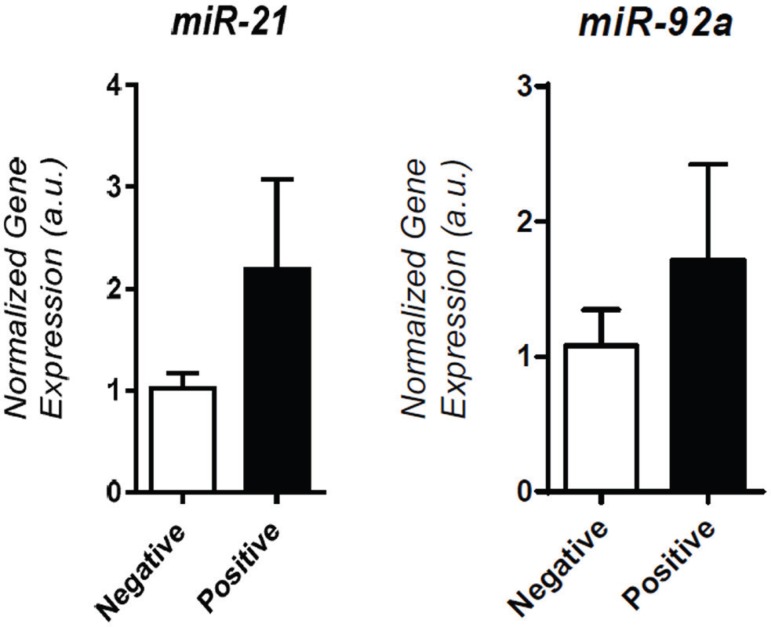
Expression of miR-21 and miR-92a in the Positive and Negative Implantation groups

Expression of four of the seven tested miRNAs was detected in spent medium from pooled samples. Three miRNAs were differently expressed between the groups. A significant increased expression of miR-142-3p was seen in the negative implantation group (*P*<0.001) (Fig 1). A non-statistical difference was observed in the expression of two other miRNAs (miR-21 and miR-92a) (Fig 2). A highly specific amplification of mature miRNAs, including miR-142-3^-^, was observed when ten non-redundant samples were tested for miRNA in individual samples of culture medium (data not shown).

## DISCUSSION

The importance of miRNAs in embryonic stem cell lines and embryo development in several species has been proven. Several studies have described dynamic changes in miRNA expression in gametes and during early embryo development of mammalian species (Tang *et al*., 2007; Tesfaye *et al*., 2009; Hossain *et al*., 2012; Mondou *et al*., 2012; Abd El Naby *et al*., 2013). However, little is known about human embryo miRNA expression. The results from this pilot study demonstrate that increased expression of miR-142-3p might be associated with implantation failure in ICSI cycles.

Bioinformatic analysis of miR-142-3p predicted targets showed its potential role on the regulation of several biological pathways and cell functions, including cell cycle inhibition and tumor suppression (Lee *et al*., 2014). However, the biological role of miR-142-3p during early human embryo development is yet to be determined.

Hergenreider *et al.* (2012) showed that cultured cells secrete miRNA into the surrounding medium. In this context, embryos developing in vitro offer a promising possibility of detecting miRNA expression in spent culture medium and identifying noninvasive biomarkers of embryo viability and implantation potential.

The main characteristics of an ideal embryonic biomarker are: (1) noninvasive assessment, (ii) stability over time, (iii) embryo specificity, and (iv) easy measurement to allow fast assessment of embryo competence. MiRNAs seem to fit it perfectly (Capalbo *et al*., 2016). In fact, the stability of miRNAs and resistance to degradation is well known (Chen *et al*., 2008; Mraz *et al*., 2009). Since miRNAs may be encapsulated in exosomes, which are small vesicles that offer additional protection from degrading enzymes (Boon & Vickers, 2013) or conjugated with macromolecular complexes (Weber *et al*., 2010), they are protected from degradation and can be detected after extended periods of time (Jung *et al*., 2010).

Rosenbluth *et al.* (2013) were the first to attempt to describe miRNA secreted from embryos in culture medium. The authors observed that the most highly expressed miRNA in euploid embryos was miR-372. Several differentially expressed miRNAs were discovered based on chromosomal status, including gender of the embryo.

Another study by Rosenbluth *et al,* (2014) detected ten miRNAs, but eight yielded false-positive signals derived from the protein supplement used in the culture medium, since miRNAs were also present in the 'blank' culture medium prior to embryo culture. The authors also looked into whether MiRNAs were differentially secreted according to embryo chromosomal status and pregnancy outcome. Higher expression levels of two miRNAs, which were not present in the blank culture medium, were associated with embryo aneuploidy (miR-191) and pregnancy failure (miR-191 and miR-372). The association between both miRNAs and pregnancy failure was found only in embryos derived from conventional IVF cycles, suggesting that sperm injection alters miRNA secretion patterns.

More recently, Capalbo *et al.* (2016) comprehensively characterized the profile of miRNAs secreted by human embryos in spent culture medium and explored whether miRNAs could be used as biomarkers of ICSI outcomes. The study revealed that two miRNAs (miR-20a and miR-30c) had higher concentration levels in the spent medium of implanted blastocysts. Both miRNAs are suggested to be involved in 23 pathways related to embryo implantation.

Several different miRNAs have been involved in the assessment of embryo competence. However, there is no consensus in the literature over this issue. The inconsistencies may be explained by the different methods used in miRNA analysis and the differences in the day of collection of spent medium; in our study spent medium was collected on day 3 from embryos achieving the blastocyst stage on day 5, while others collected spent medium on day 5. Moreover, during embryo development, there is constant synthesis and degradation of miRNAs. It has been shown that miRNAs are maternally inherited with the loss of approximately 60% between the one- and two-cell stages during the maternal zygotic transition (Tang *et al*., 2007), with an overall increase in miRNAs expression by the blastocyst stage (Yang *et al*., 2008).

miRNAs have been suggested as suitable candidates for biomarkers of embryo competence for their association with several diseases (Bernstein *et al*., 2003; Tzur *et al*., 2008; Foshay & Gallicano, 2009; Medeiros *et al*., 2011; Wang *et al*., 2012). Recent studies indicated a biological role for miRNA in controlling ovarian function (Imbar and Eisenberg, 2014), in which there is intense exchange of miRNAs between the oocyte and granulosa cells (Fiedler *et al*., 2008). Some studies have also described altered miRNA expression in patients with ovarian dysfunctions, such as polycystic ovarian syndrome (Sang *et al*., 2013; Roth *et al*., 2014) and premature ovarian failure (Yang *et al*., 2012). Moreover, poor response to controlled ovarian stimulation has also been related to altered miRNA expression (Karakaya *et al*., 2015).

In animal models, new communication systems mediating the crosstalk between the preimplantation embryo and the endometrium based on miRNA expression have been recently discovered (Sengupta *et al*., 2006; Pawar *et al*., 2013; Rosario *et al*., 2014; Chu *et al*., 2015). Specific miRNAs may act by transferring information from the blastocyst to the surrounding endometrial cells, thus altering the outcome of implantation.

This study has been affected by the following limitations: (i) a small case basis, and (ii) a limited number of analyzed miRNAs. Despite the relatively small number of embryos included in our analysis, we were able to detect significant differences in miRNA expression between the groups. We confirmed that miR-142-3^p^ is differentially expressed in human embryos that achieve the blastocyst stage according to their implantation status. This study provided additional evidence that miRNAs are secreted from human embryos into the culture medium, which makes miRNA a good candidate biomarker for embryo competence and implantation development. Future studies are required to determine whether embryo culture medium might be enriched or deprived of specific miRNAs and improve embryo development.

In conclusion, our preliminary results support the need to further explore miRNA expression in spent culture medium as a noninvasive biomarker of embryo quality and implantation potential in ICSI cycles.
